# Clinical predictors of treatment response to gabapentin in women with unexplained chronic pelvic pain

**DOI:** 10.3389/fphar.2024.1460206

**Published:** 2024-12-03

**Authors:** Lydia Coxon, Maryam Amer, Jane Daniels, Ann M. Doust, Scott C. Mackenzie, Andrew W. Horne, Katy Vincent

**Affiliations:** ^1^ Nuffield Department of Women’s and Reproductive Health, University of Oxford, Oxford, United Kingdom; ^2^ Faculty of Medicine and Health Sciences, University of Nottingham, Nottingham, United Kingdom; ^3^ Centre for Reproductive Health, Institute for Regeneration and Repair, University of Edinburgh, Edinburgh, United Kingdom

**Keywords:** chronic pelvic pain (CPP), gabapentin, treatment response, placebo, side effects, predictors

## Abstract

**Introduction:**

Chronic pelvic pain affects up to 24% of women worldwide and for up to 55% of these there is no associated pathology. Despite this there are no established treatments in this cohort. This is a secondary analysis of a randomised-controlled trial (GaPP2) to explore if there are measures which enable us to predict treatment outcome.

**Methods:**

GaPP2 recruited women with chronic pelvic pain and no identified pathology and compared the response to gabapentin and placebo. This analysis used variables collected at baseline including validated questionnaires. Binary logistic regression was used to create models to explore whether baseline variables predicted treatment response. Treatment response was determined using 30% reduction in average pain intensity, 30% reduction in worst pain intensity and the Patient Global Impression of Change (‘marked’ or ‘very marked’ improvement) individually. We also explored whether baseline variables predicted the occurrence of side-effects (dizziness, visual disturbances and drowsiness).

**Results:**

Using the Patient Global Impression of Change questionnaire, we found a significant binary logistic regression (*p* = 0.029, explaining 31% of the variance), with those with lower worst pain intensity (odds ratio (OR) of 0.393, 95% CI [0.217, 0.712]), lower bladder symptom score (OR = 0.788, CI [0.628, 0.989]), and higher mental component quality of life score (OR = 0.911, CI [0.840, 0.988]), more likely to have ‘marked’ or ‘very marked’ improvement when treated with gabapentin. We could not identify predictors of experiencing side-effects to gabapentin. However, we did find predictors of these in the placebo group (binary logistic regression (*p* = 0.009) and explained 33% of the variance). Worse mental health (OR = 1.247, CI [1.019, 1.525]) and lower baseline pain interference (OR = 0.687, CI [0.483, 0.978]) were associated with having side effects, whilst the use of hormones reduced the risk of experiencing side effects (OR = 0.239, CI [0.084, 0.676]).

**Discussion:**

Researchers and clinicians are increasingly aware of the importance of personalised medicine and treatment decisions being driven by knowledge of what treatments work for whom. Our data suggests an important role of the Patient Global Impression of Change in clinical trials as it may better reflect balance between symptoms reduction and side-effects and therefore be more useful in clinician-patients joint decision making.

## 1 Introduction

Chronic pelvic pain (CPP) is common and has a significant impact on quality of life and work productivity (Zondervan et al., 1999; Latthe et al., 2006; Daniels and Khan, 2010; Lamvu et al., 2021). Whilst it can be associated with underlying pathology (e.g., endometriosis) (Zondervan et al., 2018; Horne and Missmer, 2022), for more than half of those undergoing laparoscopy no obvious cause will be identified (Daniels and Khan, 2010). Unfortunately, there are no established treatments available for unexplained CPP in women. Given the increasing awareness that mechanisms underlying chronic pain are frequently similar no matter what the associated pathology or where the pain is perceived to arise from (Treede et al., 2019), there has been a move towards using pain-focussed therapies for all chronic pain conditions even if no condition-specific evidence exists.

One example of this is gabapentin, for which there is evidence of effectiveness in neuropathic pain (Wiffen et al., 2017), as well as studies showing that gabapentinoids affect brain function in people with chronic pain and in models of central sensitisation (Iannetti et al., 2005; Harris et al., 2013). The off-label use of gabapentin in CPP had increased with many GPs stating that they would consider prescribing gabapentin to treat CPP in men and women (Montastruc et al., 2018), although the rates appear to have started to drop following it becoming a controlled drug (Ashworth et al., 2023). To determine the efficacy and safety of this approach, a UK multicentre randomised double-blind placebo-controlled trial of the efficacy of gabapentin in women with CPP with no obvious pelvic pathology (GaPP2; Vincent et al., 2018; Horne et al., 2020) was carried out. Following feedback from public patient involvement (PPI) work, the trial had dual primary outcome measures of both average and worst pelvic pain scores, as there was no consensus amongst patients about which was more important (Vincent et al., 2018). The primary analysis of this study is already published, and the trial demonstrated no significant clinical nor statistical difference in average or worst pain scores between those treated with gabapentin compared to placebo (Horne et al., 2020). Moreover, side effects (including most commonly dizziness, visual disturbances and drowsiness) were higher in the women who had received gabapentin. These findings are clinically important because of the increased risk of suicidal behaviour, risk of dependence and possible misuse of gabapentin (Syed et al., 2023).

However, despite an overall lack of efficacy, the trial data suggest subgroups of women derive benefit. A proportion of women treated in the trial with gabapentin experienced a ≥30% improvement in worst (n = 30/124, 24%) and/or average pain (n = 44/123, 36%), representing a “moderate improvement” (Horne et al., 2020), compared to placebo (n = 21/122, 17%; n = 37/121, 31% respectively). Taking a group level approach to data analysis thereby risks denying treatment to a subgroup who may actually derive real benefit from it. In the era of personalised medicine, it is important to consider the use of clinical data collected at baseline to identify factors which predict response to treatment and/or side-effects (Edwards et al., 2016; Dworkin and Edwards, 2017).

Thus, we undertook a secondary analysis of the gabapentin trial data to identify baseline predictors of response to treatment, placebo effect and side effects.

## 2 Materials and methods

### 2.1 Study design and participants

GaPP2 was a multicentre, randomised, double-blind, placebo-controlled trial that recruited between 22nd January 2016 and 6th March 2019 across 39 UK hospital centres. Ethical approval for the trial was obtained from the UK Coventry and Warwick Research Ethics Committee (REC 15/WM/0036) and clinical trial authorisation was obtained from the Medicines and Healthcare Products Regulatory Authority. The full protocol and primary analysis have been previously published elsewhere (Vincent et al., 2018; Horne et al., 2020).

Participants were eligible if they had chronic pelvic pain (with or without dysmenorrhoea or dyspareunia) for at least 3 months. These criteria were based on the Royal College of Obstetricians and Gynaecologists (Royal College of Obstetricians and Gynaecologists, 2012) definition for chronic pelvic pain and the 2012 International Association for the Study of Pain taxonomy (Task Force, 2012), as this study design preceded the International Classification of Diseases-11 classification system (Nicholas et al., 2019). All trial participants provided written informed consent.

In addition, participants were required to be 18–50 years old, using/willing to use contraception, and have no obvious pelvic pathology at laparoscopy (e.g., macroscopic endometriosis lesions, ovarian cyst >5 cm) between 2 weeks and 36 months prior to consent. Participants were excluded for the following reasons: current or previous use of a gabapentinoid; surgery planned in next 6 months; contraindications to taking gabapentin; a malignancy; only dysmenorrhoea experienced; breastfeeding, pregnant or pregnancy planned in the next 6 months; unable/unwilling to stop taking gonadotropin-releasing hormone agonists (if taking); suspected gastrointestinal origin of pain; previous participation in GaPP1 pilot study (Lewis et al., 2016).

Prior to randomisation, eligible participants underwent a screening phase in which they were asked to report via a bespoke text messaging system their average and worst pelvic pain scores on a Numerical Rating Scale (NRS) (0 no pain to 10 worst pain imaginable), weekly for 4 weeks. Participants had to return at least three scores for both worst and average pain and at least two of the worst pain scores needed to be greater or equal to four to be randomised into the trial. Participants were then randomly assigned to receive either placebo or gabapentin in a 1:1 ratio. Participants, clinicians, and research staff were blinded to the trial group assignments throughout.

Participants were asked to take the study drug daily from the date of randomisation for 16 weeks with an initial 4-week dose escalation phase (Vincent et al., 2018; Horne et al., 2020).

### 2.2 Baseline and outcome measures

Measures of average and worst NRS scores were collected weekly via the text messaging system between weeks 13–16. The final measure of average pain was taken as a mean of the reported average pain scores; and the final measure of worst pain was taken as the maximum of the reported worst pain scores. The outcome measure of Patient Global Impression of Change (PGIC) was also collected at the end of the study, as recommended by the IMMPACT guidelines for chronic pain (Dworkin et al., 2008).

Participants were also asked to complete questionnaires prior to taking the IMP (baseline) and upon completion of treatment (week 16). The questionnaires included a variety of validated measures which can be seen in Table 1.

**TABLE 1:** Questionnaires completed at baseline (prior to taking IMP) and completion of treatment (week 16).

Concept	Measure
General quality of life with summary scores for physical component (PCS) and mental component (MCS)	Short Form (SF) – 12 (Ware et al., 1996). PCS and MCS scored from 0 to 100 with higher scores indicating better quality of life
Fatigue	Brief Fatigue Inventory (BFI) (Mendoza et al., 1999). Scored from 0–10 with higher scores meaning greater fatigue
Pain interference	Brief Pain Inventory (BPI), (Cleeland and Ryan, 1994). Scored from 0 to 10, with higher scores indicating greater interference
Psychological distress	General Health Questionnaire (GHQ) (Goldberg, 1988; Jackson, 2006). Scored 0 to 12, with greater scores indicating greater psychological distress
Pain related cognitions such as rumination, magnification and helplessness	Pain Catastrophizing Questionnaire (PCQ) (Sullivan et al., 1995). Scored from 0 to 52, with greater scores relating to worse catastrophizing
Bladder related symptoms including ‘pain’ and ‘bother’	Pelvic Pain and Urgency/Frequency (PUF) ‘Pain’, ‘Bother’ and ‘Total’ scores. PUF symptom score ranges from 0 to 23, PUF bother score ranges from 0 to 12, and PUF total score ranges from 0 to 35; a score greater than 12 is indicative of clinically significant symptoms

### 2.3 Analysis

All statistical analysis to determine predictors was carried out on SPSS version 29 and with figures created on Graphpad Prism version 10.

To test the differences in baseline variables between treatment groups (gabapentin vs. placebo) a Mann-Whitney U Test was used. The analyses of the gabapentin and placebo treatment groups were conducted separately and were not directly compared. The three outcome measures of 30% reduction in average pain, 30% reduction in worst pain and PGIC, were used to determine whether an individual was a ‘responder’ or ‘non-responder’: using a reduction in 30% for the NRS scores for each of worst and average pain (in line with IMMPACT guidelines, to reflect at least moderate clinically important differences), and ‘marked’ or ‘very marked’ improvement on PGIC.

The most common side-effects (dizziness, drowsiness, and visual disturbances) were also investigated for potential predictors. In the primary analysis there was a significant difference in occurrence of these side-effects between placebo and gabapentin. Side-effect data was collected at weeks 4–5 and 8–10, either in person when the IMP was dispensed or by telephone.

Binary logistic regression models were created to identify predictors of treatment response for each of the three outcome measures and for side-effects, for both the gabapentin and placebo groups separately. For the outcome measures where a significant regression model was not found, Mann-Whitney-U tests were also run, comparing the baseline variables between ‘responders’ and ‘non-responders’ or those with and without side effects. A Chi-Squared test was used to test the use of hormonal medication as a potential predictor of treatment response and side effects.

## 3 Results

### 3.1 Demographics

The demographics of the trial participants are detailed in Table 2. Participants were balanced for each of the baseline variables and there were no significant differences in any baseline variable, between the two treatment groups, as described in the published primary analysis (Horne et al., 2020).

**TABLE 2:** Demographics of gabapentin and placebo groups. Shown are number and (percentage), and mean (standard deviation) as appropriate. Adapted from (Horne et al., 2020). PCQ = Pain Catastrophizing Questionnaire; BFI = Brief Fatigue Index; MCS = Mental Component Score (from Short-Form-12); PCS = Physical Component Score (from Short-Form-12); PUF = Pelvic Pain and Urgency/Frequency Questionnaire; GHQ = General Health Questionnaire; NRS = Numerical Rating Scale.

	Gabapentin (n = 153)	Placebo (n = 153)
Age (years)	30.5 (7.7)	30.1 (8.6)
BMI (kg/m^2^)	27.1 (5.7), 151	27.8 (5.9), 150
Menstruating	109 (71%)	108 (71%)
Dysmenorrhoea	100 (65%)	100 (65%)
GHQ total score	4.6 (3.7)	4.7 (3.7)
Current use of sex hormones	99 (65%)	99 (65%)
Pain score during periods	7.7 (1.6), 103	7.6 (1.7), 103
PUF symptom score	9.7 (4.1)	10.0 (4.5), 148
PUF bother score	5.3 (2.6)	5.4 (2.8), 150
PUF total score	15.0 (6.3)	15.5 (7.0), 147
painDETECT score	13.4 (6.6)	13.0 (6.5)
PCS	38.9 (9.5)	40.6 (9.2)
MCS	40.2 (10.7)	40.2 (11.5)
BFI	5.2 (2.4)	5.1 (2.4)
Pain interference	3.5 (2.8)	3.8 (2.8)
PCQ	27.3 (13.4)	26.3 (13.2)

### 3.2 Outcome groups

Using the outcome measures of average NRS, worst NRS and PGIC as grouping variables, participants were classified as ‘Responders’ or ‘Non-responders’ to the treatment they were given. The proportions of participants falling into each group can be seen in Table 3 with 30% reduction in average NRS giving the greatest number of ‘responders’ for both placebo and gabapentin groups. Baseline variables of responders and non-responders are shown in Tables 4, 5 for responders defined by average and worst NRS respectively.

**TABLE 3:** Number and (percentage) of participants classified as Responders according to each of the outcome measures for both the gabapentin and placebo groups. Percentages are given out of those in the treatment group. NRS = Numerical Rating Scale, PGIC = Patient Global Impression of Change.

Outcome measure	Responders (Gabapentin) n (N, %)	Responders (Placebo) n (N, %)
Average NRS Score (30% reduction)	44 (123, 35.8%)	37 (121, 30.6%)
Worst NRS Score (30% reduction)	30 (124, 24.2%)	21 (122, 17.2%)
PGIC (‘”marked’” or “very marked” improvement)	34 (112, 30.4%)	22 (108, 20.4%)

**TABLE 4:** Shows baseline variables compared in ‘responders’ and ‘non-responders’ groups defined using average NRS. Shown are median and (IQR) as well as Z and *p* values from Mann-Whitney tests and estimate of difference and 95% confidence intervals (CI). PCQ = Pain Catastrophizing Questionnaire; BFI = Brief Fatigue Index; MCS = Mental Component Score (from Short-Form-12); PCS = Physical Component Score (from Short-Form-12); PUF = Pelvic Pain and Urgency/Frequency Questionnaire; GHQ = General Health Questionnaire; NRS = Numerical Rating Scale.

Average pain	‘Responders’ median (IQR)	‘Non-responders’ median (IQR	Responders vs. non-responders		
Predictor Variable			p	Difference	95% CI
Age	24.2 (21.1–34.7)	28.6 (23–36.3)	0.075	2.3	−0.35, 5.74
BMI	25.5 (22.9–30.9)	26.3 (24.0–32.4)	0.507	0.77	−1.46, 2.77
**Average NRS**	4.5 (3.5–6)	5.3 (4.3–6.8)	0.026*	0.75	0, 1.5
Worst NRS	9 (8–9)	9 (8–9)	0.433	0	0, 1
Dysmenorrhea NRS	6 (0–8)	6 (0–8)	0.897	0	−1, 1
**GHQ**	2 (0–5)	5 (2–7)	0.002**	2	1, 3
PUF Symptom	9 (7–11)	9 (6–13)	0.787	0	−2, 2
PUF Bother	5 (4–7)	5 (3–7)	0.882	0	−1, 1
PUF Total	14 (12–19)	15 (9–22)	0.81	0	−3, 3
painDETECT	11 (6–14)	13 (9–18)	0.055	3	0, 5
PCS	41.4 (37.0–49.3)	41.1 (34.1–46.6)	0.16	−2.83	−6.43, 1.06
**MCS**	46.7 (38.2–52.3)	38.5 (0.6–45.9)	0.004**	−6.84	−11.27, −2.13
**Pain Interference**	3.6 (1.4–6)	5.9 (4–7)	0.004**	1.57	0.57, 2.71
**BFI**	4.3 (2.8–6.4)	5.2 (3.6–7)	0.025*	1.11	0.11, 2.11
**PCQ**	21 (14–29)	26 (18–41)	0.014*	7	2, 12

Significant differences between the groups are highlighted in bold.

**TABLE 5:** Shows baseline variables compared in ‘responders’ and ‘non-responders’ groups defined using worst NRS. Shown are median and (IQR) as well as Z and p values from Mann-Whitney tests and estimate of difference and 95% confidence intervals (CI). PCQ = Pain Catastrophizing Questionnaire; BFI = Brief Fatigue Index; MCS = Mental Component Score (from Short-Form-12); PCS = Physical Component Score (from Short-Form-12); PUF = Pelvic Pain and Urgency/Frequency Questionnaire; GHQ = General Health Questionnaire; NRS = Numerical Rating Scale.

Worst pain	‘Responders’ median (IQR)	‘Non-responders’ median (IQR)	‘Responders’ vs. ‘non-responders’		
Predictor Variable			p	Difference	95% CI
Age	26.5 (21.6–40.4)	27.4 (22.8–25.1)	0.86	0.35	−3.87, 3.74
BMI	25.0 (22.6–30.9)	26.4 (24.0–31.9)	0.36	1.25	−1.45, 3.52
Average NRS	4.5 (3.8–6.0)	5.4 (4–6.8)	0.061	0.75	0, 1.67
Worst NRS	9 (8–9)	9 (8–9)	0.595	0	0, 1
Dysmenorrhea NRS	6 (0–8)	6 (0–8)	0.961	0	−1, 1
**GHQ**	2 (0–4)	6 (0–8)	0.012*	2	0, 4
PUF Symptom	8 (7–13)	9 (6.3–13)	0.754	0	−2, 2
PUF Bother	5 (3–7)	5 (3–7.8)	0.744	0	−1, 2
PUF Total	13 (11–20)	14.5 (10–21)	0.706	1	−3, 4
painDETECT	12 (8–14)	12 (9–18)	0.261	2	−1, 5
PCS	42.3 (39.0–48.0)	41.1 (34.1–47.3)	0.084	−3.87	−8.0, 0.52
MCS	46.7 (36.6–52.3)	39.2 (32.6–46.7)	0.13	−4.25	−10.16, 1.49
**Pain Interference**	3.6 (1.7–6)	5.8 (3.3–7.2)	0.010**	1.71	0.43, 3
BFI	4.4 (3.1–5.8)	5.2 (3.5–7)	0.07	1	−0.11, 2.11
**PCQ**	17 (13–31)	25.5 (18.3–39)	0.018*	7	1, 13

Significant differences between the groups are highlighted in bold.

**FIGURE 1:**
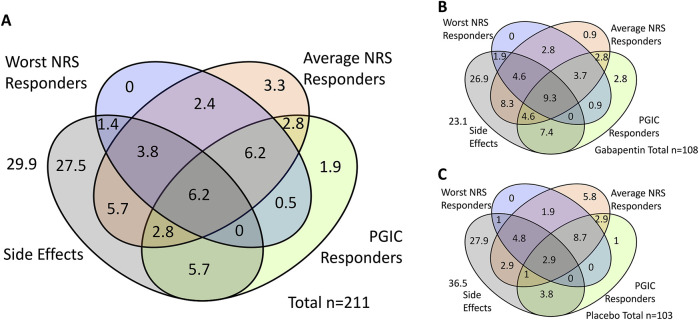
Overlap of ‘responders’ according to three outcome measures and experience of side effects. Shown are percentages. **(A)** shows the total cohort, **(B)** shows those in the gabapentin group and **(C)** shows those in the placebo group. NRS = Numerical Rating Scale, PGIC = Patient Global Impression of Change.

We found that n = 112 (50.7%) experienced the side-effects investigated (dizziness, drowsiness and/or visual disturbances). Figure 1 highlights the overlap between the treatment response defined with the three outcome measures, and the experience of side-effects.

### 3.3 Predictors of gabapentin response

If gabapentin response was defined using average NRS scores, we found no significant predictors of ‘responders’, nor did we see any significant differences between ‘responders’ and ‘non-responders’ for any of the measures.

When exploring ‘responders’ defined by worst NRS scores, our models did not identify any significant predictors, however pain interference was significantly different between the ‘responders’ (median 4.6, IQR [1.7–5.7]) and ‘non-responders’ (median 5.7, IQR [3.3–6.9]) (Z = −2.632, *p* = 0.008, difference estimate = 1.3, 95% confidence interval 0.3–2.4).

**FIGURE 2:**
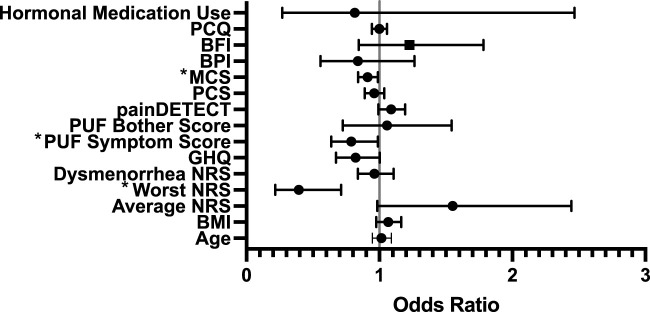
Forest Regression Plot showing results of the binary regression model assessing predictors of treatment response to gabapentin amongst PGIC responders. Mental Component Score (MCS, from Short Form-12) (*p* = 0.025), PUF = Pelvic Pain and Urgency/Frequency Questionnaire Symptom Score (*p* = 0.04) and Worst Numerical Rating Scale (NRS) (*p* = 0.02) were identified as significant predictors. PCQ = Pain Catastrophizing Questionnaire; BFI = Brief Fatigue Index; MCS = Mental Component Score (from Short-Form-12); PCS = Physical Component Score (from Short-Form-12); PUF = Pelvic Pain and Urgency/Frequency Questionnaire; GHQ = General Health Questionnaire; NRS = Numerical Rating Scale.

With ‘responders’ defined using PGIC, a significant (*p* = 0.029) model was created, which explained 31% of the variance, with increased likelihood of being a responder being associated with lower NRS scores for worst pain at baseline (odds ratio (OR) of 0.393, 95% CI [0.217, 0.712]), lower PUF symptom score (OR = 0.788, CI [0.628, 0.989]), and higher MCS (OR = 0.911, CI [0.840, 0.988]) (see Figure 2).

### 3.4 Predictors of placebo response

For placebo, no significant model was found for predicting response for any of the outcome measures. However, we did find significant differences in measures between ‘responders’ and ‘non-responders’. Firstly, when ‘responders’ were defined using average NRS, there were significant differences between ‘responders’ and ‘non-responders’ for GHQ, MCS, pain interference, fatigue and pain catastrophizing (see Table 3). Secondly, when ‘responders’ were defined using worst NRS, we found significant differences between ‘responders’ and ‘non-responders’ for pain interference, pain catastrophizing score and psychological distress (GHQ). We did not find any significant differences when using the PGIC to define ‘responders’ and ‘non-responders’.

### 3.5 Predictors of side-effects

**FIGURE 3:**
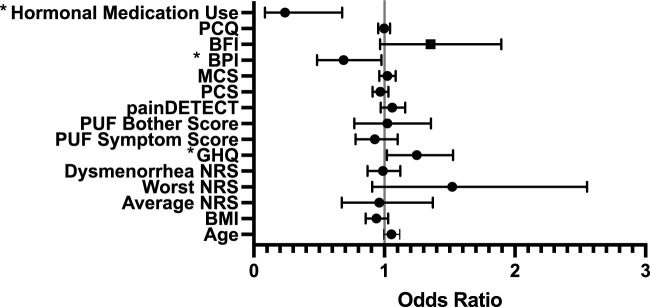
Forest Regression Plot showing results of the Binary regression model, assessing for predictors of side effects to placebo. GHQ, Pain Interference and hormones were all identified as predictors of side effects to placebo. BPI = Brief Pain Inventory which measures Pain Interference; PCQ = Pain Catastrophizing Questionnaire; BFI = Brief Fatigue Index; MCS = Mental Component Score (from Short-Form-12); PCS = Physical Component Score (from Short-Form-12); PUF = Pelvic Pain and Urgency/Frequency Questionnaire; GHQ = General Health Questionnaire; NRS = Numerical Rating Scale.

There were no significant model of predictors, or differences between those that did and did not experience side-effects for gabapentin. For side effects to placebo, our model was statistically significant (*p* = 0.009) and explained 33% of the variance. Worse mental health (GHQ OR = 1.247, CI [1.019, 1.525]) and lower baseline pain interference (OR = 0.687, CI [0.483, 0.978]) were associated with having side effects, whilst the use of hormones reduced the risk of experiencing side effects (OR = 0.239, CI [0.084, 0.676]) (see Figure 3).

## 4 Discussion

### 4.1 Main findings

In this secondary analysis of the data from our published UK wide multicentre trial of the efficacy of gabapentin for the management of unexplained chronic pelvic pain in women, we aimed to identify predictors of response to and side effects from gabapentin (and placebo). For gabapentin, there were no baseline measures that predicted a 30% reduction in pain score, nor side-effects. However, we were able to identify factors that predicted response based on the Patient Global Impression of Change (PGIC). For placebo, we found no baseline factors which predicted response, neither using PGIC nor 30% reduction in pain scores. Nevertheless, we were able to determine factors that predicted side effects from placebo.

These findings are important in the context of how we interpret placebo-controlled trials as has been discussed previously (Tracey, 2010; Vase and Wartolowska, 2019). The assumption of placebo-controlled trials is that the trial drug has an effect which is the sum of the placebo effect plus the pharmacological effect. However, if different underlying mechanisms are generating the reported outcome for the placebo effect compared to the treatment effect, then this is an oversimplification (Vachon-Presseau et al., 2018; Winkelman and Jaros, 2018; Hartmann et al., 2023; Wang et al., 2024). The fact that we do not see the same predictors of response in gabapentin compared to placebo would support this view. It is interesting however, that we can predict response to gabapentin using the PGIC questionnaire as the outcome, given that this measure combines both side-effects and treatment response.

### 4.2 Clinical implications

Our data suggest that gabapentin may be more effective for those with lower pain scores for worst pain, lower bladder (PUF) symptom scores and higher mental component scores in the quality of life measure (MCS in SF-12), all of which are less severe on their relative scales. However, as this is exploratory analysis, this would need to be validated in a larger cohort before they could have direct implications on clinical practice.

This study, and those like it, highlight the shift in research to try to identify subgroups of individuals who respond differently to treatments (Dworkin and Edwards, 2017; Gewandter et al., 2019). This shift from the search for a ‘one-size-fits-all’ treatment in chronic pelvic pain is very welcome, and we believe will lead to a future with more individualised treatment options, which will result in better treatment results for individuals with chronic pelvic pain, an area which still has very limited effective treatments. This change has been seen in chronic pelvic pain associated with endometriosis, in which there is increasing research exploring pain mechanisms and potential stratifying tools, particularly tools to explore nociplastic (Orr et al., 2022; Raimondo et al., 2023; Till et al., 2023) and neuropathic (Coxon et al., 2021; 2023) mechanisms. Studies have shown in endometriosis-associated pain that measures of nociplastic pain are related to treatment outcomes, particularly surgical outcomes (As-Sanie et al., 2021; Orr et al., 2023). A recent separate secondary analysis of the GaPP2 cohort used genome-wide association analyses to identify genetic variants associated with gabapentin response, including both analgesic efficacy and side effects. This study identified a loci in the gene Neuregulin 3 (*NRG3*) which was associated with a 30% reduction in worst and/or average NRS scores following gabapentin treatment (Mackenzie et al., 2024). These findings, although in need of validation, support the potential for genotyping to stratify gabapentin treatment to those expected to benefit. Combining genetic data with baseline questionnaire data could enhance response prediction, though the effectiveness of such multimodal strategies remains unknown and requires further investigation.

Importantly, this study highlights the utility of the PGIC as a measure of treatment efficacy. This outcome is recommended as part of the IMMPACT recommendations for clinical trial design and is a measure which may better translate to clinical practice where patients and clinicians have to weigh up the ‘cost’ in terms of side effects and ‘benefit’ in terms of pain relief and improvement in quality of life (Dworkin et al., 2008; Edwards et al., 2016). Often treatments that have been found to reduce pain in clinical trials, do not have the same improvement rate in the real-world (Sheldrick, 2023; Vollert et al., 2023). When clinicians and patients are deciding on the best treatment option(s), there is naturally discussion about the balance between pain reduction and potential side-effects, and what an individual considers most important. The PGIC inherently takes into account an individual’s cost-benefit assessment, therefore those that report ‘marked’ improvement in this measure are likely to be those that would report real-world improvement.

### 4.3 Predictors of the placebo effect

Additionally, this study highlights the need for greater exploration of the underlying mechanisms of the placebo effect, and how this can be harnessed in clinical treatment. Given that clinical trials often compare to placebo, it is also important that any predictors of placebo response are equally distributed between the treatment and placebo groups. In line with other studies, this study suggests that factors which predict placebo response may not be the same as those that predict treatment response (Freeman et al., 2015; Vachon-Presseau et al., 2018; Winkelman and Jaros, 2018; Hartmann et al., 2023; Wang et al., 2024).

Placebo and nocebo are both complicated neuropsychological phenomena, that positively and negatively (respectively) affect treatment response, both in terms of reduction in pain and experience of side effects(Caliskan et al., 2024). In the last decade there has been an increase in research around placebo and nocebo mechanisms (Vase and Wartolowska, 2019) and how these mechanisms can be harnessed to improve clinical outcomes (Caliskan et al., 2024). Several studies investigating the role of different substances have shown the complexity of neurobiological mechanisms of the placebo response, these have included highlighting the role of the release endogenous opioids (Zubieta et al., 2005; Eippert et al., 2009), the effects of giving oxytocin (Kessner et al., 2013) and vasopressin (Colloca et al., 2016) to increase the placebo response. Studies utilising neuroimaging to try to better understand placebo mechanisms have shown both structural and functional differences in multiple brain areas in placebo ‘responders’ compared to non-responders (Tracey, 2010; Vachon-Presseau et al., 2018; Zunhammer et al., 2021; Crawford et al., 2023; Hartmann et al., 2023).

Expectation of treatment outcome is strongly influenced by previous treatment experiences, meaning that if someone has more previous experience of unsuccessful treatments, they are likely to have lower expectations of treatment response to a new treatment, and therefore are less likely to be a ‘responder’ to the treatment (Basedow et al., 2023). Thus, prior treatment experiences may be an important factor in predicting response to placebo as well as side-effects, which we in this study have not collected. However, this may explain why being on hormonal medication reduces side-effects to placebo as those who experienced side-effects previously to hormonal medication would, we hypothesise, be less likely to take them during the trial. It is also possible that those that had previously experienced side-effects to one treatment (e.g., hormones), may therefore, to an extent, expect to experience side-effects to other treatments (Bingel and Placebo Competence Team, 2014). Conversely, we do not see this relationship when looking at side-effects to gabapentin itself, suggesting that there are additional/different mechanisms giving rise to symptoms, which would be plausible given the pharmacological profile of gabapentin and known side-effects (Wiffen et al., 2017).

In our secondary analysis of the trial data, we were not able to produce significant models to predict ‘responders’ to placebo, using any of our variables (although some were shown to be different between ‘responders’ and ‘non-responders’). In line with other studies those in our ‘non-responder’ group had worse baseline psychological distress and pain catastrophizing (Darnall and Colloca, 2018; Wang et al., 2022). Several studies have explored predictors of placebo response, using a variety of different phenotypic measures (Horing et al., 2014; Freeman et al., 2015; Branco et al., 2023). For example, it has been shown that social emotions and behaviours, such as helping behaviour, are increased in those that respond to placebo (Hartmann et al., 2023). In another study, interoceptive awareness and the trait of ‘openness’ were shown to be predictors of placebo response (Vachon-Presseau et al., 2018). Lower quality of sleep has also been shown to reduce the placebo response (Wang et al., 2024). These were not measures that we had included in our outcome set however.

## 5 Limitations

This is a secondary analysis of previously published trial data (Horne et al., 2020) and thus it is not designed or powered to investigate the role of potential predictors of treatment response in a definitive way. Therefore, this exploratory analysis, whilst interesting cannot be directly translated into clinical practice without further investigation in a prospective cohort.

Additionally, there are measures which may have a role in predicting treatment response, which are not captured within the trial dataset. This could include the number and type of previous treatments tried, expectations in relation to the treatment, general health behaviours such as exercise and diet, individual priorities in what they want from treatments (reduction in average pain, worst pain, pain interference, etc.), as well as measures which as discussed have been shown to predict placebo response. This is also true of predictors of side-effects, for which rates were high, and may relate to gastrointestinal alterations which were not included in our baseline measures.

This trial recruited women with unexplained CPP (including individuals with no obvious pelvic pathology at laparoscopy) therefore the findings cannot be extrapolated to chronic pelvic pain related to pathologies such as endometriosis, or CPP that has not been investigated via a surgical approach. Whilst unexplained CPP is common, it is important that primary or secondary analysis of the results from this trial are not taken out of this context.

## 6 Conclusion

Given that we do not see the same predictors of treatment response to gabapentin and placebo, our secondary analysis supports previous work suggesting different mechanisms may generate the same reported outcome in placebo and active treatment arms. In this study, we cannot predict the likelihood of side-effects or treatment response using the (NRS) pain score derived outcomes for gabapentin. However, we can identify predictors of the PGIC, with less severe symptom scores associated with greater likelihood of treatment response. This highlights the utility of the PGIC as an outcome measure as participants themselves weigh up cost-benefit to self, reflecting the clinical situation of joint decision making.

## Data Availability

The data analyzed in this study is subject to the following licenses/restrictions: Patient-level data will be made available. Requests will be assessed for scientific rigor before being granted. Data will be anonymized and securely transferred. A data sharing agreement may be required. Requests to access these datasets should be directed to Andrew Horne andrew.horne@ed.ac.uk.
